# Research priorities for Indian psychiatry

**DOI:** 10.4103/0019-5545.69201

**Published:** 2010-01

**Authors:** Vikram Patel

**Affiliations:** Department of Epidemiology and Population Health, London School of Hygiene and Tropical Medicine Sangath, Public Health Foundation of India, Alto Porvorim, Goa, India

**Keywords:** Research priorities, research capacity, implementation science, epidemiology

## Abstract

This article summarises the findings of recent priority setting exercises for psychiatric research and of a mapping of research capacity and resources in south Asia. The priorities for research in the region, as in other developing countries, are related to ‘implementation’ science, i.e. the field of inquiry investigating acceptable and affordable methods of delivering effective treatments for mental disorders, which aims to help close the large treatment gap. “Discovery” research which aims to strengthen our understanding of the nature of mental disorders through well-designed epidemiological and descriptive clinical studies, and expand the armamentarium of effective treatments by mapping and evaluating indigenous approaches to mental health care is also an important priority. However, research capacity and resources in the region are scarce and need strengthening by action from diverse stakeholders including the Indian Psychiatric Society.

The primary role for research in the context of medicine is to generate knowledge which will ultimately lead to improvement in health. In the field of psychiatry, our primary concern is the mental health of the population we serve. The reality of mental health care in India is bleak: A population of over a billion is served by less than 4000 psychiatrists, the majority of whom are based in cities and concentrated in a few states; about 20,000 inpatient beds, 80% of which are located in 40 odd mental hospitals most of which were built decades ago; and the almost total absence of community or primary care based mental health services with the notable exceptions of a few NGO and public initiatives scattered around the country.[1] It is not surprising, then, that this situation leads to a large “treatment gap”. At best, no more than half of all persons with serious mental disorders in India receive the care we know can help improve their quality of lives; amongst those who do, the care is often entirely medication based, and for many who find their way to mental hospitals, characterized by loss of dignity and frank human right abuse.[2] This situation is by no means unique to India; indeed it is mirrored in most low and middle income countries.

The large treatment gap was the primary motivation for the landmark *Lancet* series on global mental health published in 2007. As part of that series, a systematic research priority setting exercise was carried out in the context of the call to action to scale up services for people with mental disorders based on the twin principles of evidence and protection of human rights.[3] The detailed findings of this exercise were published recently.[4] This exercise focused on four mental disorders: Schizophrenia and other major psychotic disorders; major depressive disorder and other common mental disorders; alcohol abuse and other substance abuse disorders; and child and adolescent mental disorders. Across all four conditions, priority research questions related to delivery issues: i.e. How can we deliver treatments know to be effective in a cost-effective manner and through routine health care systems. Child and adolescent mental disorders, and alcohol use disorders were given the highest priority, an indication of the relatively large treatment gaps for these conditions and the paucity of contextually appropriate evidence on these conditions. The lowest rankings were given to discovery *r*esearch options, for example those that proposed the development of new interventions and technologies, new drugs, vaccines or pharmacological agents.

This exercise proposed that in the context of the large treatment gaps, and with a time frame of the next 10 years, investments would be best placed in health policy and systems research in order to provide guidance about how to increase access to cost-effective treatments. In particular, the emphasis in these priorities was to better understand how to deliver what we know works in an affordable manner, what has been referred to as ‘implementation’ science.[5] A particularly important research question in this context is to develop and evaluate mental health interventions for delivery by non-specialist health workers, a strategy referred to as ‘task-shifting’ in current global health parlance. There is now a growing evidence base on the effectiveness of such interventions from South Asia, and these need to be evaluated and replicated.[6–9]

The Manas trial, the largest trial for any mental disorder in any low/middle income country, is an example of such an ambitious enterprise;[10] its results on the clinical benefits and cost-effectiveness of a lay health worker led intervention for depression and anxiety disorders in primary care in Goa will be published in 2010. The COPSI trial, which is evaluating a community based intervention for people with schizophrenia based on a model which was developed and field-tested in Madhya Pradesh,[7,11] is currently in progress in three sites in India (Goa, rural Tamil Nadu and Satara) and represents a unique partnership between NGOs and private psychiatrists.

A key challenge to implement these research priorities is that of capacity and resources to implement research. Despite a large medical school network, our region has barely contributed to the mental health research literature,[12] especially in the field of intervention research.[13]

A recent initiative, led by the Global Forum for Health Research and the World Health Organization (WHO), aimed to assess research resources and capacity and describe the current research agendas, priority-setting, and impact of research on policy. Six regional teams implemented a common protocol; the South Asian team was led by the author and included partners in Pakistan, Sri Lanka, Bangladesh and Nepal. The research had three components. The first component was carrying out a standardized search of Medline and Psychinfo databases for all indexed research literature from the respective countries. This was supplemented by a search of local journals, databases and grey literature. A database comprising the names of active researchers in the region with their details was compiled. The second component consisted of a survey of all the lead authors of articles identified through the search. The questionnaire elicited a range of information on research including research resources, methods, priorities and funding. In the final phase of the project we carried out in-depth interviews with key informants to elicit their responses on some of the salient findings of the survey and their views on the interface of policy and research.

Findings of the global survey have been published recently.[14,15] The findings specifically related to South Asia are summarized here.

From the search of the indexed literature, over a five-year period for India (1998 to 2003) and 10 years for the other countries (1993 to 2003), 899 articles from South Asia were identified. India accounted for 4 out of 5 papers published in indexed journals from the region. The next most important contributors were Pakistan and Sri Lanka. In general, the proportionate distribution of articles in the indexed literature is roughly equivalent to the proportion of the population of South Asia for each country; Sri Lanka seems to be an exception with a relatively higher proportion of articles in the indexed literature. No articles were identified from three countries: Maldives, Afghanistan and Bhutan. Less than half of the indexed articles were published in journals with an ISI Impact Factor. The mean IF of these articles was moderate (2.81, 95% CI 2.48-3.14; estimated on the IFs in 2005). Eight authors accounted for the lead authorship of nearly 10% of the total output of the region. We also searched the local and non-indexed literature from various databases, journals and libraries in each country and identified a total of 475 articles.

From this research publication database, we were able to identify and locate addresses for 691 researchers of whom 223 responded to the questionnaire survey. Majority of the respondents were psychiatrists (46%). The majority worked in hospitals, research organizations or universities with relatively few in private sector organizations. Only about 1/3 of the respondents reported belonging to a research network (either local or international). With regard to access to the literature on mental health, 87% of respondents reported having access to internet resources but mostly to free databases. Only 20% of respondents had access to paid internet sites with full text articles. In all, 70% researchers reported that they had not received any kind of research fellowship or grants in the five years prior to the survey. The most important obstacle hindering research was lack of funds (52%) followed by lack of time. Although the vast majority of respondents (87%) had some training in at least one of the major research methods, there did not appear to be enough of a critical mass in any one research methodology.

Research priorities were elicited through two routes: First, by asking direct questions on priorities and priority-setting process; second, by eliciting details about the three most recent mental health research projects. The latter yielded data on 468 research projects. There was a high level of concordance between reported priorities and actual research topics. The most commonly cited priorities epidemiological studies of the burden of disease and risk factors as the first priority for future mental health research (48%). This was followed by health systems (17%) and social science research (16%). This rank ordering was identical for the actual research projects. Similarly, in terms of mental health conditions, the priorities for future research, and the commonest research project subjects, were anxiety-depression followed by psychosis and substance abuse. Women, children and adolescents and the poor were identified as the three most important marginalized populations for mental health research.

Overall, there were very small numbers of research publications. In the five years prior to the survey, only 36% of the respondents had greater than five local publications (and 8% had more than five in international journals), with no significant differences by country. Only 22% of researchers reported one or more conference presentations a year. Researchers reported a reasonable amount of media coverage with 61% noting that their research findings were covered in the local newspaper. Print media was much more likely to be the mode of coverage than electronic media. Despite this coverage, only about 27% of respondents reported having had their research publicized in the form of direct policy materials for policy makers.

This study concluded that the South Asian region suffers from very inadequate mental health research resources in terms of both financial support (funding for individuals and institutions) and professional support (e.g. involvement in research networks, access to the literature, training in research methodology). Though some examples of research impacting policy are available, in general there is little interface between research and policy. The project has identified the major mental health research priorities and resources for the region, which are shared by both researchers and stakeholders; this agenda needs to be implemented through a concerted effort to build research capacity, improve the communication of findings to a wide range of stakeholders (in particular policy makers), and advocate for research resources in the region.

Indian psychiatry has a critically important role to play in furthering the research agenda identified through these two systematic exercises. Elsewhere, I have argued that the role of psychiatry in developing countries needs to fundamentally change from focusing on individual clinical care to a much broader set of public health skills, including research.[16] Without such a radical revision of the scope of our work, the treatment gap will not reduce. In order to achieve this transformation, there is a need for much stronger training in research methodology, preferably integrated with post-graduate training. The very limited impact of the MD dissertations on mental health policy and practice in India reflects on the relatively weak mentorship currently available in most medical schools.

The recent renaissance in public health training, best exemplified by the new Public Health Foundation of India’s networks of public health schools around the country,[17] offers exciting new opportunities to strengthen research training of psychiatrists. There are a growing number of training programs in public mental health and research (see www.globalmentalhealth.org for a full list); these include short courses such as the Development and Evaluation of Complex Interventions and the Leadership in Mental Health courses, run by Sangath in Goa (www.sangath.com). The Indian Psychiatric Society could forge an alliance with these initiatives to run research training courses, and promote innovation in the use of mixed-methods teaching curricula where part of the course work is done by distance thereby minimizing the amount of time trainees need to spend in a class-room.

We also need to look beyond delivery questions. The truth is that even the best treatment in psychiatry has limited impact on mental health and related social outcomes. There is a renewed need for *discovery* of more effective treatments, both pharmacological and psychological, which can increase the armamentarium of modern psychiatry. The South Asian region is home to traditional systems of medicines which have long addressed mental disorders. A comprehensive research program which documents and evaluates the pharmacological agents used by these systems, and which evaluates locally appropriate psychological and social interventions for mental disorders, holds out the exciting promise of discovering altogether new treatments for use in biomedical psychiatry.

We need to be inspired by the re-discovery of artemisinin, the wonder drug for malaria which was used as a traditional Chinese herbal remedy for malaria since 200 BC (http://en.wikipedia.org/wiki/Artemisinin). Yoga, for example, could be an important contribution to evidence-based treatments for mental disorders globally.[18] Further, we are still a long way from understanding the etiology of most mental disorders; researching population in diverse cultural and social contexts is a critically important step to building our knowledge base on the nature of mental disorders.

Thus, Indian psychiatry needs to invest in research at both the delivery and discovery ends of the research spectrum,[19] and to ally with public health training initiatives in the region to strengthen research capacity. The ultimate mission of research in psychiatry in our region must be both to close the treatment gap and to strengthen the scientific evidence base for the discipline of psychiatry.
